# Chemical Composition and Biological Activities of *Croton sertanejus* Essential Oil Against *Aedes aegypti* and Preliminary *Toxicity* in *Mus Musculus*

**DOI:** 10.1002/cbdv.71759

**Published:** 2026-09-27

**Authors:** Daniel Lobo Sousa, Morgana Maria do Carmo Barbosa, Rômulo Carlos Dantas da Cruz, Ivone Antonia de Souza, Rosilene Aparecida de Oliveira, Débora Cardoso da Silva, Simone Andrade Gualberto, Eduardo Carvalho Lira, Janaína Silva de Freitas

**Affiliations:** ^1^ Biochemistry and Molecular Biology State University of Southwestern Bahia (UESB) Itapetinga BA Brazil; ^2^ Environmental Sciences State University of Southwestern Bahia (UESB) Itapetinga BA Brazil; ^3^ Pharmaceutical Sciences Department of Antibiotics Federal University of Pernambuco Recife PE Brazil; ^4^ Pharmacology Department of Antibiotics Federal University of Pernambuco Recife PE Brazil; ^5^ Chemistry Department of Exact and Technological Sciences State University of Santa Cruz Salobrinho BA Brazil; ^6^ Veterinary Sciences Research Center in Applied Chemistry Department of Exact and Natural Sciences (DCEN)/(UESB) Itapetinga BA Brazil; ^7^ Pharmaceutical Sciences Research Center in Applied Chemistry Department of Exact and Natural Sciences (DCEN)/(UESB) Itapetinga BA Brazil; ^8^ Physiology Department of Physiology and Pharmacology Federal University of Pernambuco Cidade Universitária Recife PE Brazil; ^9^ Biochemistry, Department of Exact and Natural Sciences (DCEN)/(UESB) Itapetinga BA Brazil

**Keywords:** biorepellent, mosquito, mouse, natural products, terpenoids

## Abstract

The objective was to evaluate the essential oil (EO) from *Croton sertanejus* leaves against *Aedes aegypti*, analyzing spatial repellency (SR), topical effective concentration (EC), EC protection time, oviposition deterrence, and preliminary toxicity in *Mus musculus*. Additionally, the chemical composition of the oil was characterized. Results showed a significant SR of 0.2 starting from 1 mg/cm^2^. The ECs required to repel 50% and 99% of the mosquitoes were 3 and 109 mg/cm^2^, respectively. The EC of 109 mg/cm^2^ provided 64.8% protection against landings within 360 min and 100% protection against blood‐feeding for 150 min. The EO significantly reduced oviposition at water volumes of 20 mL (beginning at 2.5 mg/mL) and 500 mL (beginning at 0.005 mg/mL); however, no significant effect was observed at 1000 mL. No mortality occurred in *Mus musculus*. However, at a dose of 2000 mg/kg, a significant reduction in brain weight was observed, while at 300 mg/kg, an increase in lung and kidney weights occurred. At 2000 mg/kg, there were also elevated levels of aspartate aminotransferase and alanine aminotransferase, alongside a reduction in creatinine. Regarding the chemical composition, eucalyptol was the main component. Therefore, these findings underscore the need for further validation.

## Graphical abstract

This study investigated the chemical composition and biological potential of the essential oil from the newly described species *Croton sertanejus*. The results demonstrated efficacy in repellency and oviposition deterrence against the *Aedes aegypti* mosquito, coupled with low mammalian toxicity, suggesting the potential of this oil as a viable alternative for vector control.

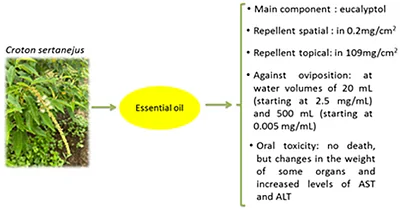

## 1 Introduction

Exposure to vector organisms constitutes one of the major global public health challenges. A prominent example is the female mosquito of the species *Aedes aegypti* (Linnaeus, 1762), the primary urban vector for the four dengue virus serotypes, the zika virus, and chikungunya. These viruses can cause severe morbidity and, in more critical cases, may be fatal [1].

The use of repellents represents one of the primary methods for protection against mosquito bites. These agents are classified into two categories: spatial and topical. Spatial repellents act by releasing active substances into the air to repel mosquitoes or deter them from entering a specific environment. Conversely, topical formulations are applied directly to the skin to deter these vectors from the human body [2]. The synthetic active ingredients present in spatial and topical repellents include 2,3,5,6‐tetrafluorobenzyl (1R,3S)‐3‐(2,2‐dichlorovinyl)‐2,2‐dimethylcyclopropanecarboxylate, also known as transfluthrin, and N,N‐diethyl‐3‐methylbenzamide (DEET), respectively [3, 4].

Regarding transfluthrin, the *Ae. aegypti* mosquito has shown reduced susceptibility to its repellent action. As this is a synthetic pyrethroid compound, concerns exist regarding its efficacy in regions where vector resistance to this chemical class is prevalent [5]. Furthermore, pyrethroid‐resistant *Ae. aegypti* may also exhibit decreased sensitivity to the repellent DEET [5], demonstrating the importance of seeking alternative repellents. Additionally, human health risks must be considered, including fatal accidental ingestion, potential alterations in sex hormones, adverse impacts on bone health in children and adolescents, and hyperuricemia [6, 7, 8].

However, the temporal and spatial distribution of arboviruses transmitted by *Ae. aegypti* shows that the use of repellents alone has not been sufficient to protect against the vector, further highlighting the importance of seeking complementary methods [9]. Accordingly, the present research also proposed oviposition deterrence as a complementary protection method against *Ae. aegypti*, which consists of discouraging mosquitoes from laying eggs in potential breeding sites [10].

Essential oils (EOs) are among the alternative substances with potential as repellents and oviposition deterrents [10]. EOs are volatile organic compounds produced and stored in different plant structures and organs, such as leaves [11, 12]. Among the advantages of EOs compared to synthetic repellents are their numerous chemical compounds, making it difficult for the *Ae. aegypti* mosquito to develop resistance [12].

EOs can be found in plant species of the genus *Croton*, including the leaves of *Croton sertanejus* (Sodré & Silva, 2022) [13]. Although species of the genus *Croton* have been evaluated as repellents against *Ae. aegypti*, few studies have been conducted [14], demonstrating that this is an underexplored genus for this purpose. With the aim of investigating new species of the genus against the mosquito *Ae. aegypti*, this study evaluated the repellent activity and oviposition deterrence of the EO of a recently described plant, *C. sertanejus* [13].

Furthermore, we investigated the chemical composition of *C. sertanejus* EO, providing the first report on its characterization and biological potential. While searching for new plant‐derived bioactive substances targeting the vector is crucial, toxicological studies are indispensable to establish safe concentrations [15]. Therefore, since this study evaluated oviposition deterrence in artificial containers that mimic human‐use water reservoirs, determining acute oral toxicity remains essential.

In this context, the objective was to evaluate the EO from *C. sertanejus* leaves against *Ae. aegypti*, by analyzing spatial repellency, topical effective concentration (EC), EC protection time, oviposition deterrence, and preliminary toxicity in *Mus musculus* (Linnaeus, 1758). Additionally, the chemical composition of the oil was characterized.

## 2 Results and Discussion

Chemical analysis of *C. sertanejus* EO identified 19 out of 33 detected compounds, mostly sesquiterpenes. Among the identified compounds, eucalyptol was the major constituent (13.8%), followed by bicyclogermacrene (8.95%), 𝛾‐cadinene (6.22%), and spathulenol (6.16%). Further details regarding the chemical composition are presented in Table 1.

**TABLE 1:** Chemical composition of the essential oil from the leaves of *Croton sertanejus*.

Constituents	^a^ *C. sertanejus* (%)	^b^ EKRI	^c^ LKRI
α‐Pinene	4.55	938	939
Sabinene	1.47	976	975
<o‐> Cymene	0.58	1025	1026
Eucalyptol	13.8	1030	1031
1,6‐Octadien‐3‐ol, 3,7‐dimethyl	2.32	1034	NI
trans‐dihydro‐β‐Terpineol	1.53	1097	1138
Terpinen‐4‐ol	1.53	1180	1177
α‐Terpineol	1.50	1192	1188
< *δ‐*> Elemene	0.73	1342	1338
α‐ Copaene	0.62	1383	1376
β‐Bourbonene	0.43	1393	1388
NI	4.88	1397	NI
NI	11.44	1433	NI
α‐Humulene	2.71	1466	1454
allo‐Aromadendrene	1.58	1473	1460
𝛾‐Cadinene	6.22	1493	1513
β‐Selinene	0.64	1498	1490
trans‐Muurola‐4(14),5‐diene	0.86	1504	1493
Bicyclogermacrene	8.95	1510	1500
NI	0.71	1524	NI
NI	1.19	1532	NI
NI	0.73	1544	NI
NI	2.34	11591	NI
Spathulenol	6.16	1595	1578
NI	7.46	1601	NI
NI	4.38	1623	NI
NI	1.49	1628	NI
NI	4.73	1655	NI
Cubenol	0.86	1660	1646
α‐Cadinol	1.28	1669	1654
NI	0.95	1671	NI
NI	1.21	1702	NI
NI	0.48	1721	NI
Total identified	57.57		
Hydrocarbon monoterpenes	15.78		
Oxygenated monoterpenes	21.05		
Hydrocarbon sesquiterpenes	47.36		
Oxygenated sesquiterpenes	15.78		

^a^ Substances listed in order of elution on a FactorFour VF‐5 ms capillary column.

^b^ Experimental Kovats retention indices.

^c^ Literature Kovats retention indices [16].

^d^ NI, Not identified.

These findings suggest that the *C. sertanejus* EO is less volatile, which is expected given the higher molecular weights of its predominant compound class [17]. To our knowledge, the chemical composition of the EO from *C. sertanejus* leaves has not been previously documented.

The EO of *C. sertanejus* exhibited spatial repellent activity against *Ae. aegypti*, and a one‐way analysis of variance (ANOVA) indicated significant differences among the groups (F = 3.08, df = 5, *p* = 0.016). The Games‐Howell post‐hoc test, interpreted via bootstrapping, revealed significant activity (*p* < 0.05) at concentrations of 1 mg/cm^2^ (AI = 0.2 ± 0.11), 2.5 mg/cm^2^ (AI = 0.29 ± 0.14), and 5 mg/cm^2^ (AI = 0.47 ± 0.08) compared to the Tween 80 negative control, which presented an AI of −0.07 ± 0.08. These results are summarized in Table 2.

**TABLE 2:** Spatial activity index of the essential oil from the leaves of *Croton sertanejus* against *Aedes aegypti* females.

Treatments	Concentrations mg/cm^2^	^a^SAI ± ^b^SE	Repellency (%)
*C. sertanejus*			
	5	0.47^a^ ±0.08	55.5
	2.5	0.29^a^ ^c^ ±0.14	52.5
	1	0.2^a^ ^c^ ±0.11	51.5
	0.5	0.20^a^ ^cd^ ±0.12	50.5
	0.2	−0.02^b^ ^cd^ ±0.13	^***^
Tween 80			
	5	−0.07^b^ ^d ^± 0.08	^***^

^a^ Spatial Activity Index.

^b^ Standard error. Means were submitted to a one‐way analysis of variance (ANOVA) (*p* < 0.05), with normality adjusted by the bootstrapping method (1.000 resamplings, with a bias‐corrected and accelerated 95% confidence interval). Mean values followed by different letters differ significantly by Games‐Howell post‐hoc test (*p* < 0.05).

*** Indicates that no repellency percentage was observed. Ten independent replicates were evaluated per concentration, with *n* = 20 mosquitoes per replicate. Two replicates of each treatment were evaluated simultaneously per day. The trials were conducted in a climate controlled room at 27°C and 70% relative humidity.

The results demonstrate that spatial repellent action is concentration‐dependent, suggesting that values below 1 mg/cm^2^ may fail to confer significant protection. These findings corroborate the scarce literature regarding the spatial repellent potential of species from the genus *Croton* against *Ae. aegypti*. Teixeira et al. [18] demonstrated that the EO of *Croton argyrophyllus* (Kunth) leaves in the highest formulation, at 5%, showed spatial repellency against *Ae. aegypti* with an average SAI of 0.23 ± 0.21. Sousa et al. [12] also observed spatial repellency against *Ae. aegypti* in the highest formulation of *C. tetradenius* EO at 300 mg/cm^2^, with an average SAI of 0.36 ± 0.08. However, in the present study, the EO of *C. sertanejus* at lower concentrations exhibited higher SAIs.

As reported by Sousa et al. [12], the spatial repellency of certain EOs against *Ae. aegypti* may stem from the activation of antennal chemoreceptors and olfactory sensory neurons, structures responsible for the mosquito's olfactory navigation. In the present study, because there was no direct contact between the mosquitoes and the EO treated filter paper in the test chamber, it is believed that these same sensory mechanisms are responsible for the spatial repellency of the *C. sertanejus* EO. Furthermore, it is suggested that the spatial repellent activity of *C. sertanejus* EO may be associated with a synergistic interaction, given that the spatial repellent activity of isolated eucalyptol, evaluated by Liu et al. [19] against *Ae. aegypti*, showed only 30% repellency a value lower than the results of the present study, as shown in Table 2.

Regarding the EC for topical use, the estimated concentrations to repel 50% and 99% of mosquitoes were 3 mg/cm^2^ and 109 mg/cm^2^, respectively (Table 3).

**TABLE 3:** Effective concentration 50% and 99% of the essential oil from the leaves of *Croton sertanejus* against *Aedes aegypti* female mosquitoes within three minutes of exposure.

Treatment	Effective concentration mg/cm^2^				
*C. sertanejus*	^a^EC 50(%)	Confidence Interval	EC 99(%)	Confidence Interval	^b^χ^2^ (^c^DF) ^d^ *p*
	3	(1 ‐ 7)	109	(80 ‐ 195)	86.921(33) 0.000

^a^ Effective concentration.

^b^ Chi‐square.

^c^ Degrees of freedom.

^d^ *p*‐value. Effective concentrations were estimated using PROBIT regression analysis with a 95% confidence level (*p* < 0.05). Five concentrations were evaluated in the same cage, with 7 independent replicates and *n* = 50 mosquitoes per replicate. The experiment was conducted over a single day (08:00 to 12:00) in a controlled environment (27°C and 70% relative humidity). No adjustment procedure was required because the probit is a generalized linear model for binary data and does not rely on traditional linear assumptions [20].

The findings suggest that *C. sertanejus* EO holds potential as a topical repellent against *Ae. aegypti*.This finding corroborates the study by Sousa et al. [12], who also observed the efficacy of EOs from the genus *Croton* as topical repellents using a membrane system. Furthermore, Sousa et al. [12] reported that repelling 50% and 99% of *Ae. aegypti* mosquitoes with a *C. tetradenius* EO formulated in a non‐ionic emulsion requires concentrations of 14.1 mg/cm^2^ and 163.4 mg/cm^2^, respectively. Nevertheless, in the present study, the EO of *C. sertanejus* exhibited lower EC_50_ and EC_99_ values, as shown in Table 3.

The EC_99_ equivalent to 10.9% is consistent with commercial concentrations involving the synthetic repellent DEET, and is among the lowest concentrations commercially permitted. In Brazil, for example, DEET formulations typically contain less than 10% for children and above 30% for individuals over 12 years of age [21]. However, the EC_50_ corresponding to 0.3% is considered the lowest among commercial concentrations of DEET, which may be due to the estimated lower percentage of repellency.

Sousa et al. [12] discussed the advantages of conducting topical repellency assays using a membrane system. The authors highlighted that this type of test standardizes both the application surface area and blood temperature, besides meeting safety requirements by not exposing human volunteers to potentially toxic substances. Accordingly, considering that the toxicity profile of *C. sertanejus* EO remains unknown, the present research opted to adopt the same methodology.

Regarding protection time, EC99 provided protection against *Ae. aegypti* landings. At 30 min, Welch's one‐way ANOVA indicated a significant difference among the groups (F(3, 11) = 7.3), (*p* = 0.006). Over the same period, the Games–Howell post‐hoc test revealed that the mean repellency of *C. sertanejus* EO (99.4 % ± 0.3) differed significantly from the negative controls: cream‐treated tulle (85.4 % ± 3.7) and untreated tulle (86.8 % ± 3.7) (*p* < 0.05). However, no significant difference was observed compared to the standard repellent DEET, which provided (99.1 % ± 0.8) protection (*p *> 0.05).

At 90 min, the EO provided 97.4% ± 0.8 protection and did not differ significantly from DEET, which maintained 99.1% ± 0.8 protection until the end of the experiment (*p *> 0.05). However, both treatments were significantly more effective than the negative controls at all time points (*p* < 0.05). The negative controls exhibited a decrease in protection over time, with no significant differences among them (*p *> 0.05). The results can be observed in Table 4

**TABLE 4:** Cumulative percentage of repellency and protection time of *Croton sertanejus* leaf essential oil against *Aedes aegypti*.

	Treatments and concentration. Cumulative repellency ± ^a^SE				
Time (min)	*C.sertanejus* 109 mg/cm^2^	DEET 109 mg/cm^2^	^b^TWC	^c^TWIC	^d^Welch's ANOVA F value (^e^DF_1_.DF_2_) *p* value
30	99.4^a^ ± 0.3	99.1^a^ ± 0.8	85.4^b^ ± 3.7	86.8^b^ ± 3.7	7.3 (3.11.0) 0.006
60	98.8^a^ ± 0.5	99.1^a^ ± 0.8	70.86^b^ ± 4.6	70.8^b^ ± 6.1	16.7 (3.11.7) 0.000
90	97.4^a^ ± 0.8	99.1^a^ ± 0.8	58.5^b^ ± 7.4	55.8^b^ ± 8.8	15.8 (3.12.0) 0.000
120	94.8^a^ ± 1.6	99.1^b^ ± 0.8	48.8^c^ ± 10.3	44.5^c^ ± 10.5	15.5 (3.11.2) 0.000
150	93.4^a^ ± 1.5	99.1^b^ ± 0.8	40.0^c^ ± 11.0	32.0^c^ ± 12.7	19.3 (3.11.3) 0.000
180	91.4^a^ ± 1.9	99.1^b^ ± 0.8	35.4^c^ ± 10.9	28.8^c^ ± 12.2	23.4 (3.11.1) 0.000
210	88.5^a^ ± 2.1	99.1^b^ ± 0.8	28.8^c^ ± 10.0	26.2^c^ ± 11.8	30.8 (3.10.9) 0.000
240	88.0^a^ ± 2.3	99.1^b^ ± 0.8	23.1^c^ ± 9.7	23.7^c^ ± 11.5	34.8 (3.10.8) 0.000
270	84.8^a^ ± 3.5	99.1^b^ ± 0.8	17.7^c^± 9.5	22.0^c^ ±10.9	39.9 (3.10.4) 0.000
300	79.4^a^± 4.3	99.1^b^ ± 0.8	16.2^c^±9.4	19.1^c^± 10.4	46.2 (3.10.3) 0.000
330	71.4^a^± 6.8	99.1^b^ ± 0.8	13.4^c^±8.8	17.1^c^± 9.4	52.8 (3.10.2) 0.000
360	64.8^a^ ± 7.7	99.1^b^ ± 0.8	11.7^c^± 7.7	14.0^c^ ± 8.9	68.3 (3.10.2) 0.000

^a^ Standard error.

^b^ Tulle impregnated with cream.

^c^ Tulle without cream.

^d^ One‐way Analysis of Variance adapted by Welch's test.

^e^ Degrees of freedom. A normality adjustment using the bootstrapping method (1.000 resamples, with a bias‐corrected and accelerated [BCa] 95% confidence interval) was also applied to the one‐way ANOVA (*p* < 0.05). Means within the same row sharing different letters differ significantly according to the Games‐Howell post hoc test (*p* < 0.05). Each treatment had seven independent replicates, with *n* = 50 mosquitoes per replicate. One replicate of each treatment was evaluated simultaneously each day (08:00 and 13:00) under controlled environmental conditions (27°C and 70% relative humidity).

The results suggest that, under the evaluated conditions, *C. sertanejus* EO exhibits repellent potential against *Ae. aegypti*. Similarly, Sousa et al. [12] reported the protective efficacy of *Croton* EO, noting that *C. tetradenius* (EC_99_ of approximately 200 mg/cm^2^) provided 95% protection against landings for 30 min, maintaining 84% efficacy after 240 min. Comparatively, the EC_99_ of *C. sertanejus* EO was lower and demonstrated superior protection over the same period.

Chen et al. [22] demonstrated that monoterpenes such as eucalyptol, the main component of *C. sertanejus* EO, strongly excite *Ae. aegypti* antennal sensilla. This suggests that the repellent action of this oil against mosquito landing is mediated by antennal chemoreceptors.

EC99 provided complete protection against blood‐feeding, with no occurrences recorded for up to 150 min. The non‐parametric Kruskal‐Wallis test indicated significant differences among groups starting at 60 min χ^2^ (3) = 9.429, *p* = 0.024. Dunn's post‐hoc test revealed that the mean ranks of EC99 did not differ significantly from those of DEET at any time point (*p *> 0.05), although DEET maintained protection throughout the experiment. Compared to the negative controls, EC99‐conferred protection differed significantly at 180 and 360 min (*p* < 0.05). At 180 min, EC99 showed a mean blood‐feeding rank of 9.36, whereas the negative controls (tulle with cream and tulle without cream) exhibited mean ranks of 20.21 and 20.43, respectively. At 360 min, EC99 yielded a mean rank of 12.64, while the negative controls showed mean ranks of 20.64 and 18.71, respectively. DEET differed significantly from the negative controls starting at 180 min (*p* < 0.05). The negative controls failed to provide protection and did not differ significantly from each other at any time point (*p *> 0.05). These results are presented in Table 5.

**TABLE 5:** Mean ranks of cumulative blood‐feeding of female *Aedes aegypti* mosquitoes as a function of the protection time of the effective repellent concentration of the essential oil from *Croton sertanejus* leaves.

	Treatments and concentration. Mean ranks of cumulative blood‐feeding (Cumulative mean blood‐feeding %)						
Time (min)	*C.sertanejus* 109 mg/cm^2^	DEET 109 mg/cm^2^	^a^TWC	^b^TWIC	Median^a^	^c^IQR (25%–75%)	Kruskal Wallis ^d^ χ^2^ (^e^DF) *p‐*value
30	12.50^*^(0.0)	12.50^*^ (0.0)	14.43^*^ (0.29)	18.57^*^ (1.4)	0.00	0.00 – 0.00	6.883 (3) 0.076
60	11.00^a^(0.0)	11.00^a^ (0.0)	16.64^a^ (1.1)	19.36^a^ (2.7)	0.00	0.00 – 0.75	9.429 (3) 0.024
90	9.00^a^, ^b^ (0.0)	9.00^a^, ^b^ (0.0)	18.36^b^ (3.1)	21.64^cb^ (5.4)	0.00	0.00 – 4.00	16.961 (3) 0.001
120	9.00^a^, ^b^ (0.0)	9.00^a^, ^b^ (0.0)	18.64^b^ (4.2)	21.36^cb^ (6.7)	0.00	0.00 – 4.00	16.648 (3) 0.001
150	9.00^a^, ^b^ (0.0)	9.00^a^, ^b^ (0.0)	18.86^b^ (5.1)	21.14^cb^ (8.7)	0.00	0.00 – 4.00	16.499 (3) 0.001
180	9.36^a^ (0.2)	8.00^a^ (0.0)	20.21^b^ (6.2)	20.43^b^ (8.7)	0.00	0.00 – 4.00	16.741 (3) 0.001
210	9.79^a^, ^c^ (0.2)	8.50^a^ (0.0)	20.43^b^, ^c^ (7.1)	19.29^c^ (9.4)	0.00	0.00 – 5.5	14.809 (3) 0.002
240	9.79^a^, ^c^ (0.2)	8.50^a^ (0.0)	20.43^b^ (8.0)	19.29^cb^ (11)	0.00	0.00 – 7.50	14.789 (3) 0.002
270	9.29^a^, ^c^ (0.2)	8.00^a^ (0.0)	21.64^b^ (8.5)	19.07^cb^(12.5)	0.00	0.00 – 8.00	17.303 (3) 0.001
300	10.07^a^, ^c^ (0.5)	7.50^a^ (0.0)	21.57^b^ (9.4)	18.86^cb^(13.4)	1.00	0.00 – 9.50	16.321 (3) 0.001
330	11.07^a^, ^b^ (1.4)	6.00^a^ (0.0)	20.79^b^ (11.0)	20.14^b^ (16.2)	2.00	0.00 – 11.50	17.216 (3) 0.001
360	12.64^a^ (2.8)	6.00^a^ (0.0)	20.64^b^ (13.7)	18.71^b^ (19.4)	3.00	0.00 – 11.50	14.483 (3) 0.002

^a^ Tulle with cream.

^b^ Tulle without cream.

^c^ Interquartile range.

^d^ Chi‐square.

^e^ Degrees of freedom.

*There was no significant difference by the Kruskal‐Wallis test (*p* < 0.05). Mean ranks followed by different letters in the same row differ significantly (Dunn's post hoc test, *p* < 0.05, with Bonferroni correction for *p*‐value adjustment). Protection against blood‐feeding was evaluated in the same repellency assay over time for each period, maintaining an experimental design of seven independent replicates per treatment, with *n* = 50 mosquitoes per replicate.

These results suggest that *C. sertanejus* EO holds potential to protect against blood‐feeding by *Ae. aegypti* females. This finding corroborates the study by Sousa et al. [12], who demonstrated the efficacy of *Croton* EO in preventing mosquito blood‐feeding using a membrane system; specifically, they reported 100% protection from *C. tetradenius* EO for 240 min. However, a direct efficacy comparison between the two studies was precluded because our analysis evaluated mean ranks of blood‐feeding, whereas Sousa et al. [12] reported arithmetic means.

The absence of a significant difference between the *C. sertanejus* EO and the negative controls across different periods does not necessarily indicate a lack of efficacy, but rather the conservative nature of the Kruskal‐Wallis test when ranking data. Therefore, while the Kruskal‐Wallis test and Dunn's post hoc test do not analyze means, these measurements were provided solely to better demonstrate that the formulated EO exhibited potential in preventing blood‐feeding by *Ae. aegypti* females. The choice of a non‐parametric test in the blood meal evaluation was driven by limitations of the bootstrapping method on the data, likely caused by excess zeros.

According to Dennis et al. [23], the deterrence of blood‐feeding by female *Ae. aegypti* may be strongly associated with the stimulation of tarsal chemoreceptors. This is supported by a reduction in blood‐feeding when mosquito tarsi come into contact with potential repellents. In the present study, the EC_99_ was administered exclusively on the tulle fabric. This fabric contained perforations sufficient to allow mosquito landing, yet small enough to physically prevent the insertion of the proboscis into the artificial feeder. Therefore, it is inferred that the deterrence of blood‐feeding by female *Ae. aegypti* induced by the EO of *C. sertanejus* is mediated through this same tarsal detection mechanism.

Because EOs are highly volatile [24], the reduced protection against landings and blood‐feeding is likely attributable to this property. Furthermore, it is suggested that the topical repellent activity of *C. sertanejus* EO is mediated by synergistic interactions among its constituents, given that our results are quantitatively superior to the repellent effect of isolated eucalyptol against landing and blood‐feeding by *Ae. aegypti* females, as reported by Klocke et al. [25] and Costa et al. [26], respectively.

In the evaluation of oviposition deterrent activity, the independent‐samples *t*‐test with bootstrapping showed that the mean OAI at 5 mg/mL (OAI = −0.99) and 2.5 mg/mL (OAI = −0.95) for *C. sertanejus* EO was significantly lower than that of the negative controls (0.99 and 0.95, respectively; *p* < 0.05). All results are presented in Table 6.

**TABLE 6:** Oviposition deterrence of *Croton sertanejus* essential oil against *Aedes aegypti* in 20 mL of water.

		*t‐*test		
		^a^OAI ± ^b^SE		
EOs	Concentration mg/mL	Treatment	Control	*t*‐value ^c^(DF) *p*‐value
*C. sertanejus*	5	−0.99^a^ ± 0.00	0.99^b^ ± 0.00	−180.748 (8) 0.000
	2.5	−0.95^a^ ± 0.02	0.95^b^ ± 0.02	−45.312 (8) 0.000

^a^ Oviposition activity index.

^b^ Standard error.

^c^ Degrees of freedom. Means followed by different lowercase letters in the same row differ significantly according to the independent *t*‐test (*p* < 0.05). A normality adjustment using the bootstrapping method (1.000 resamples, with a bias‐corrected and accelerated [BCa] 95% confidence interval) was also applied. Each treatment had five independent replicates, with *n* = 20 mosquitoes per replicate. All replicates for each treatment were simultaneously arranged on laboratory benches, yet kept separate. Egg counts were performed after 96 h using a stereomicroscope. The experiment was conducted in a controlled environment (27°C and 70% relative humidity).

The results demonstrate that the EO exhibited oviposition deterrent activity, yielding significant negative values (indicating reduced egg‐laying). Furthermore, the deterrent activity of the EO in a small water volume (20 mL) was concentration‐dependent. The evaluation of the oviposition deterrent activity of EOs from species of the *Croton* genus has also been reported in the literature, such as the EO of *C. rhamnifolioides* (Pax & K.Hoffm., 1923) investigated by Santos et al. [27], as well as the species *C. zehntneri* (Pax & K.Hoffm., 1923), *C. argyrophylloides* (Müll.Arg., 1865), *C. sonderianus* (Müll.Arg., 1866), and *C. nepetaefolius* (Baill., 1864), of which the last two showed no such activity [28]. A direct comparison with these cited studies was unfeasible due to discrepancies in evaluation times and statistical methods, respectively.

At concentrations of 0.01 and 0.005 mg/mL of *C. sertanejus* EO in 500 mL of water, the independent‐samples *t*‐test showed a mean OAI of −0.75 for both treatments, which was significantly lower than the negative control value (OAI = 0.75, *p* < 0.05). These results are presented in Table 7.

**TABLE 7:** Oviposition deterrence of *Croton sertanejus* essential oil against *Aedes aegypti* in a 500 mL water volume.

		*t‐*test		
		^a^OAI ± ^b^SE		
EOs	Concentration mg/mL	Treatment	Control	*t*‐value ^c^(DF) *p*‐value
*C. sertanejus*	0.01	−0.75^a^ ± 0.09	0.75^b^ ± 0.09	−12.185 (4) 0.000
	0.005	−0.75^a^ ± 0.09	0.75^b^ ± 0.09	−12.302 (4) 0.000

^a^ Oviposition activity index.

^b^ Standard error.

^c^ Degrees of freedom. Means followed by different lowercase letters in the same row differ significantly according to the independent *t*‐test (*p* < 0.05). Each treatment had three independent replicates, with *n* = 20 mosquitoes per replicate. All replicates for each treatment were simultaneously arranged on laboratory benches, yet kept separate. Egg counts were performed after 90 h using a stereomicroscope. The experiment was conducted in a controlled environment (27°C and 70% relative humidity). No parametric adjustment procedure was applied, as preliminary verification revealed no biases or asymmetries that would justify statistical corrections.

The results demonstrate that at lower concentrations and a higher water volume (500 mL), the oviposition deterrent activity of the *C. sertanejus* EO remains significant, with the effect being concentration‐independent. To our knowledge, no prior studies have evaluated the oviposition deterrent potential of larger water volumes (500 mL) containing EOs from the genus *Croton* against *Ae. aegypti*.

In 1000 mL of water, the independent‐samples *t*‐test revealed no significant difference (*p *> 0.05) between the OAI values of *C. sertanejus* at 0.005 mg/mL (OAI = −0.18) and 0.0025 mg/mL (OAI = −0.22) and their respective negative controls (OAI = 0.23) and (OAI = 0.15), respectively. These results are presented in Table 8.

**TABLE 8:** Oviposition deterrence of *Croton sertanejus* essential oil against *Aedes aegypti* in a 1000 mL water volume.

		*t‐*test		
		^a^OAI ± ^b^SE		
Eos	Concentration mg/mL	Treatment	Control	*t*‐value ^c^(DF) *p*‐value
*C. sertanejus*	0.005	−0.18^a^ ± 0.23	0.18^a^ ± 0.23	−1.101 (4) 0.333
	0.0025	−0.22^a^ ± 0.15	0.22^a^ ± 0.15	−2.024 (4) 0.113

^a^ Oviposition activity index.

^b^ Standard error.

^c^ Degrees of freedom. Means followed by different lowercase letters in the same row differ significantly according to the independent *t*‐test (*p* < 0.05). Each treatment had three independent replicates, with *n* = 20 mosquitoes per replicate. All replicates for each treatment were simultaneously arranged on laboratory benches, yet kept separate. Egg counts were performed after 90 h using a stereomicroscope. The experiment was conducted in a controlled environment (27°C and 70% relative humidity). No parametric adjustment procedure was applied, as preliminary verification revealed no biases or asymmetries that would justify statistical corrections.

The results suggest that a more significant effect of *C. sertanejus* EO can be achieved at higher concentrations. As previously described, no studies of a similar nature involving EOs from this genus in larger water volumes (1000 mL) were found. Given that *Ae. aegypti* females select distinct oviposition sites, these assays were crucial to determine whether the mosquitoes can detect *C. sertanejus* EO diluted in different water volumes. This enabled the establishment of minimum effective and economically viable concentrations, since the species can also be stimulated to oviposit near the water surface in medium and large‐sized breeding sites [29].

A review by Mulatier et al. [30] highlights that *Ae. aegypti* uses visual, olfactory, and tarsal chemosensory systems to select oviposition sites. Since gravid females in the present study were exposed at short range to the chosen sites containing volatile substances, it is inferred that short‐range detection mechanisms mediated by chemoreceptors and olfactory cells in the antennae are involved.

This hypothesis is supported by the fact that eucalyptol, the main constituent of *C. sertanejus* EO, strongly elicits antennal olfactory sensilla in *Ae. aegypti*. However, the EO‐induced oviposition deterrence may involve synergistic interactions, as the whole oil exhibited greater efficacy than its isolated component, consistent with the findings of Klocke et al. [25].

It is well‐established that the bioactivity of EOs against *Ae. aegypti* varies as a function of the concentrations applied within a given volume [31]. Therefore, given that the present study utilized distinct absolute masses of EO and non‐proportional concentration ranges, we hypothesize that this methodological variation influenced the observed efficacy results.

**FIGURE 1:**
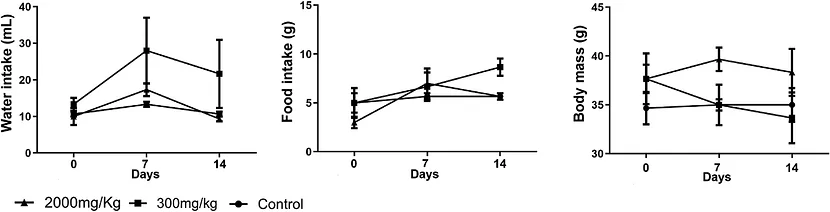
Mean consumption and body weight of mice after 14 days of oral administration of *Croton sertanejus* essential oil at doses of 2000 mg/kg and 300 mg/kg. Means were compared using two‐way repeated‐measures analysis of variance (*p* < 0,05). Vertical bars denote the standard error of the mean. Each treatment contained three independent replicates, with *n* = 1 mouse per replicate. All replicates for each treatment were simultaneously arranged on laboratory benches, yet kept separate. Animals were monitored for the first four hours and daily thereafter through day 14. The experiment was conducted in a controlled environment (24°C and 60% relative humidity). No parametric adjustment procedure was applied, as preliminary verification revealed no biases or asymmetries that would justify statistical corrections.

Regarding the preliminary acute oral toxicity test, mice treated with *C. sertanejus* EO at 300 and 2000 mg/kg showed no mortality or behavioral changes compared to the control group. The two‐way repeated‐measures ANOVA showed no significant changes in water intake (F(4.18) = 0.5741, *p* = 0.6850), food intake (F(4.18) = 1.313, *p* = 0.3027), or body mass gain (F(4.18) = 0.5833, *p* = 0.6787). These results are presented in Figure 1.

These findings suggest that *C. sertanejus* EO falls under GHS Hazard Category 5 in accordance with OECD Guideline 423 [32], which encompasses compounds with relatively low acute toxicity. Compared to the *C. tetradenius* EO reported by Carvalho et al. [33], which exhibited a median lethal dose (LD 50) of 500 mg/kg, *C. sertanejus* EO stands out as one of the least toxic within the genus.

Although a fluctuation in water intake was observed on day 7 for the 300 mg/kg group, accompanied by wide error bars, this does not necessarily indicate signs of toxicity. Rather, it reflects greater variability stemming from the sample size used, as recommended by OECD Guideline 423. The relationship between the chemical diversity of these EOs and their varying toxicities at high concentrations remains unclear. However, *C. sertanejus* EO is majorly composed of eucalyptol, a versatile therapeutic molecule known to alleviate depression, respiratory disorders, epilepsy, and diarrhea [34]. Furthermore, it exhibits anti‐inflammatory, analgesic, and pro‐apoptotic properties [34].

**FIGURE 2:**
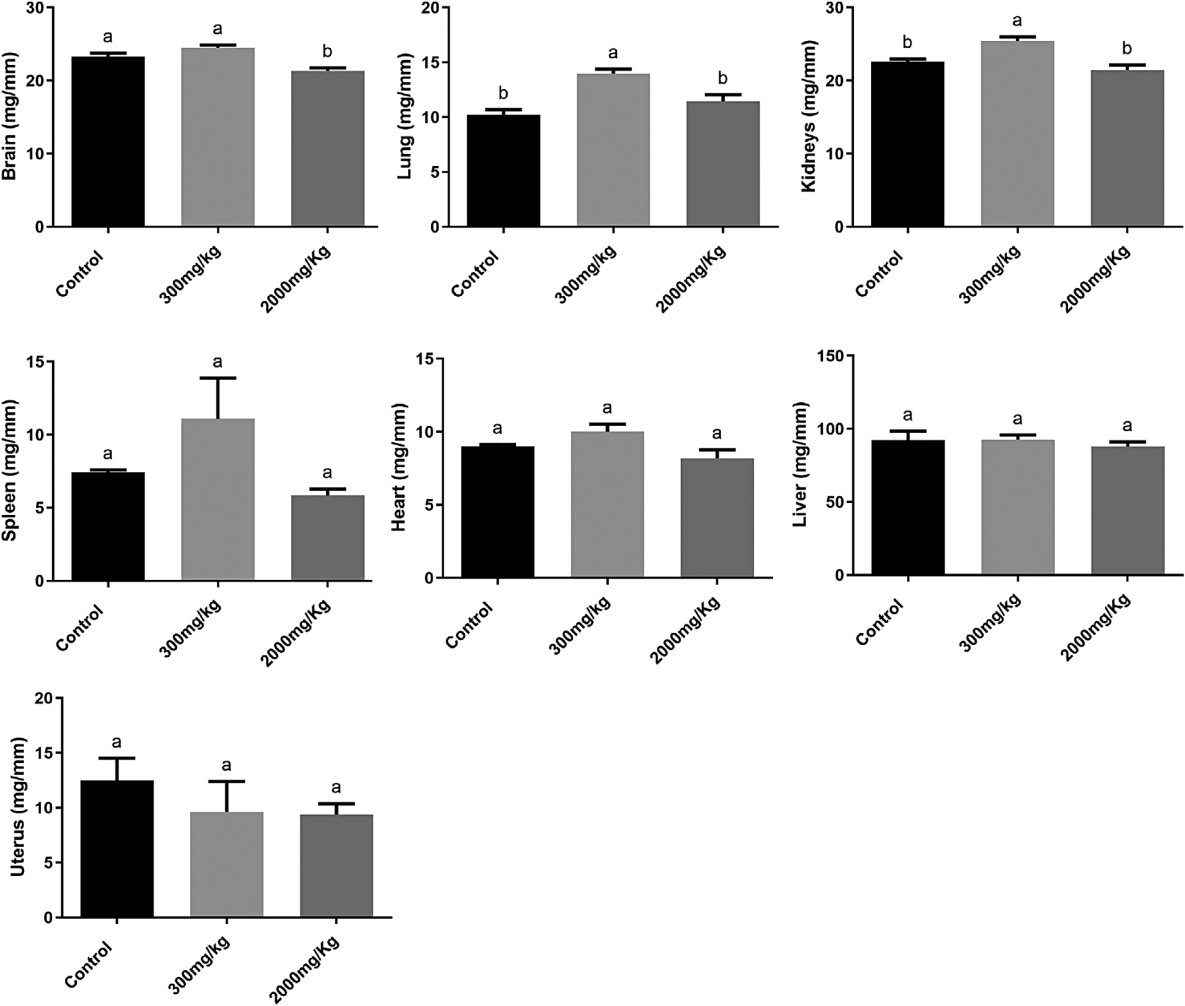
Mean organ weights of mice after 14 days of oral administration of *Croton sertanejus* essential oil at doses of 2000 mg/kg and 300 mg/kg. Means were subjected to one‐way analysis of variance (ANOVA) (*p* < 0.05). Vertical bars at the top of each column indicate the standard error of the mean. Different lowercase letters above the vertical bars indicate significant differences according to Tukey's test (*p* < 0.05). No parametric adjustment procedure was applied, as preliminary verification revealed no biases or asymmetries that would justify statistical corrections.

Regarding organ weights, one‐way ANOVA indicated no significant differences among the means of the spleen (F(2.6) = 2.751, *p* = 0.1419), heart (F(2.6) = 3.900, *p* = 0.0822), liver (F(2.6) = 0.3865, *p* = 0.6952), and uterus (F(2.6) = 0.7033, *p* = 0.5316). However, a significant difference in weight was observed only for the brain (F(2.6) = 14.05, *p* = 0.0055), lungs (F(2.6) = 14.55, *p* = 0.0050), and kidneys (F(2.6) = 12.79, *p* = 0.0069). Tukey's post hoc test showed a significant decrease (*p* < 0.05) in brain weight at the 2000 mg/kg dose (mean = 21) compared to the 300 mg/kg dose (mean = 24) and the control (mean = 23). For the lungs, there was a significant increase (*p* < 0.05) at the 300 mg/kg dose (mean = 13) relative to the control (mean = 10) and the 2000 mg/kg dose (mean = 11). Regarding the kidneys, the same 300 mg/kg) dose showed a significant increase (*p* < 0.05) compared to the control (mean = 22) and the 2000 mg/kg dose (mean = 21). These results can be seen in Figure 2.

These results suggest that *C. sertanejus* EO interacts significantly with specific organs, eliciting non‐linear responses in the lungs and kidneys.

No studies on *Croton* EOs have reported concurrent brain weight reduction and non‐linear lung or kidney responses in acutely toxic female mice. Conversely, acute toxicity trials in rats have associated decreased brain weight with neurotoxicity, and increased lung and kidney weights with inflammatory responses [35, 36].

The primary components responsible for these results remain elusive. However, given that eucalyptol, the major constituent, possesses anti‐inflammatory properties, it is hypothesized that higher doses of the EO increase the systemic bioavailability of this compound, thereby enhancing its anti‐inflammatory effects in the lungs and kidneys. The dose‐dependent effects of eucalyptol in vivo have been previously documented [37]. Although this relationship appears contradictory regarding the reduction in brain weight, the EO of *C. sertanejus* comprises other molecules, such as α‐pinene, a monoterpene linked to neurotoxic effects [38]. This compound may also have reached higher systemic concentrations following the 2000 mg/kg dose.

**FIGURE 3:**
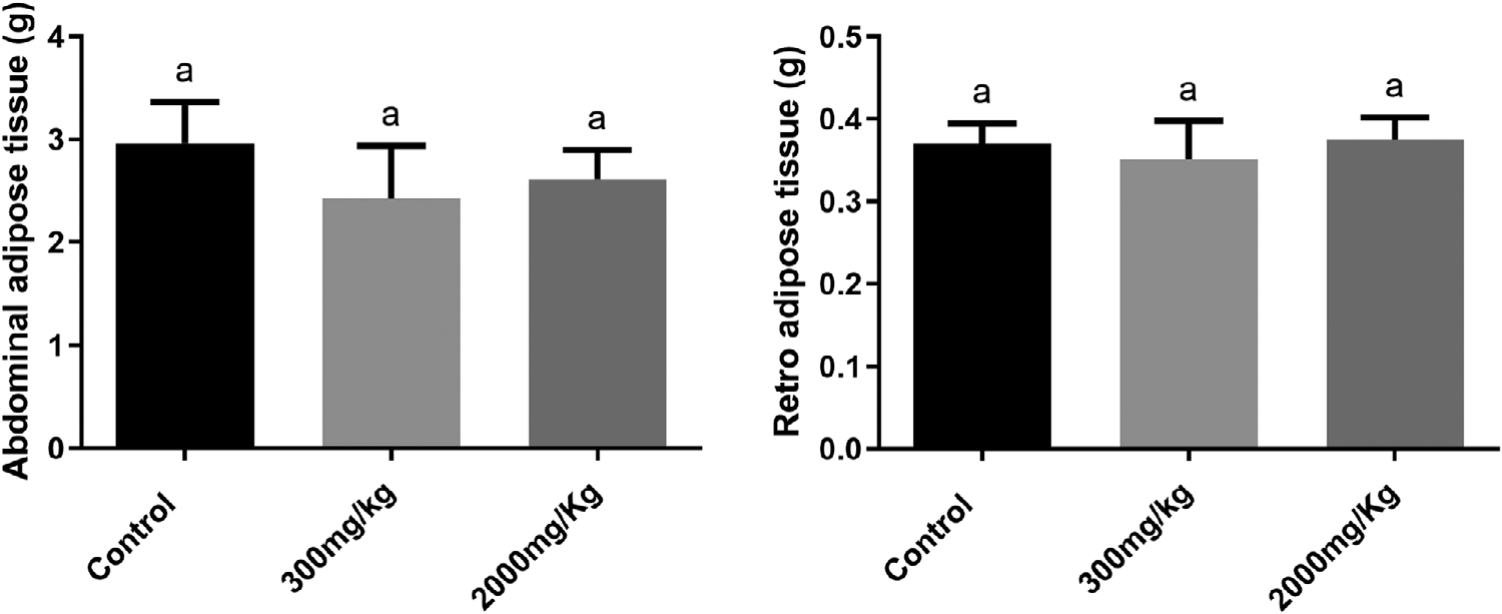
Mean weight of abdominal and retroperitoneal adipose tissue in mice following 14 days of oral administration of *Croton sertanejus* essential oil at doses of 2000 mg/kg and 300 mg/kg. Means were subjected to one‐way analysis of variance (*p* < 0.05). Vertical bars at the top of each column indicate the standard error of the mean. Means followed by different lowercase letters on the vertical bars differ significantly by Tukey's test (*p* < 0.05). No parametric adjustment procedure was applied, as preliminary verification revealed no biases or asymmetries that would justify statistical corrections.

Regarding abdominal and retroperitoneal adipose tissues, there were no significant differences in weight between the concentrations and the control (F(2.6) = 0.4429, *p* = 0.6616), and (F(2.6) = 0.1288, *p* = 0.8815), respectively, as shown in Figure 3.

Studies have shown that EOs can interact with adipose tissue [39]. However, the results of this study suggest negligible or no interaction between the EOs and these tissues. To the best of our knowledge, no previous studies have evaluated EOs from the genus *Croton* on these tissues in acute toxicity tests in female mice.

**FIGURE 4:**
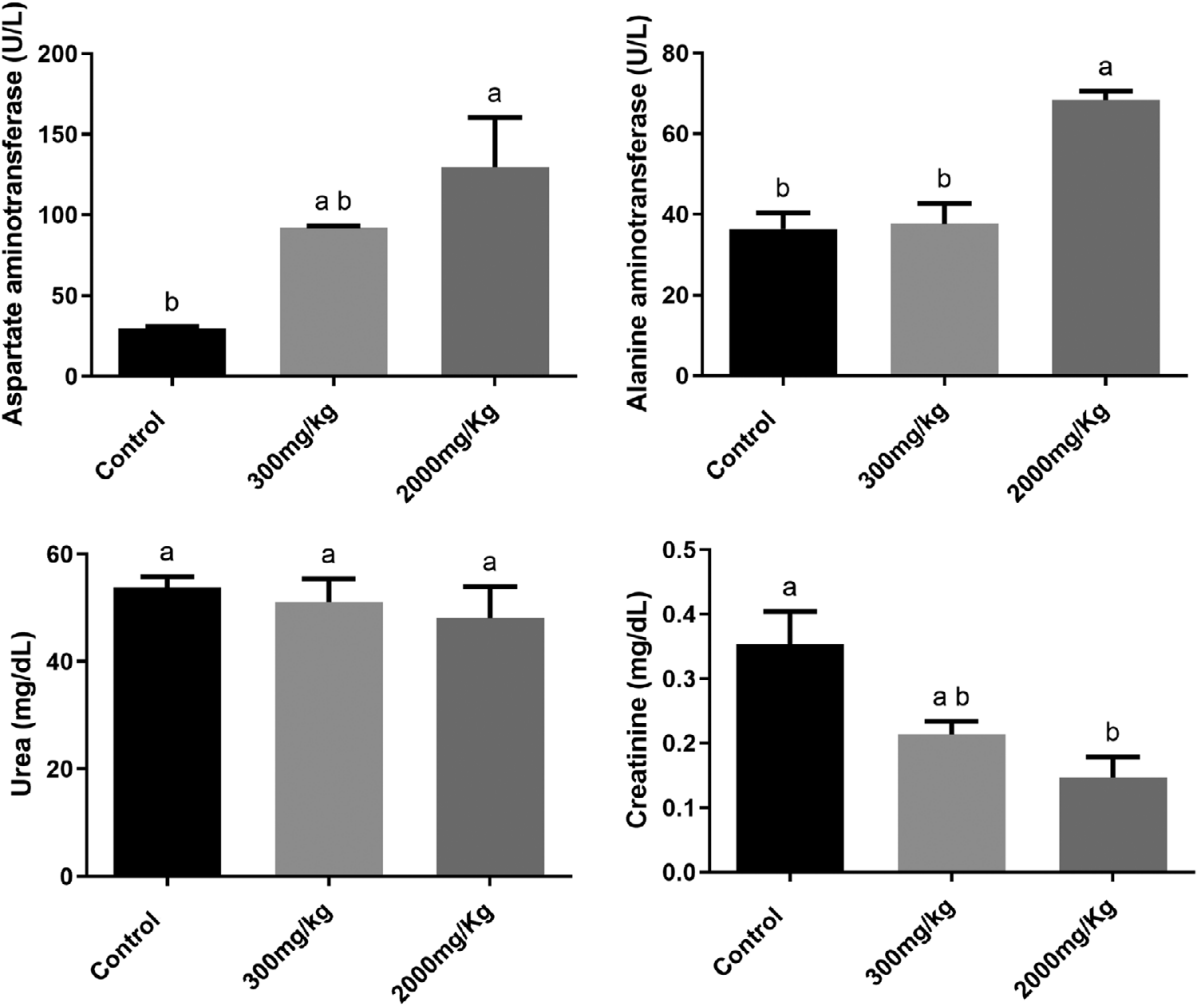
Mean biochemical parameters of the mice after 14 days of oral administration of *Croton sertanejus* essential oil at concentrations of 2000 mg/kg and 300 mg/kg. Means were subjected to a one‐way analysis of variance (*p* < 0.05). The vertical bars at the top of each column indicate the standard error of the mean. Different lowercase letters above the vertical bars indicate significant differences according to Tukey's test (*p* < 0.05). No parametric adjustment procedure was applied, as preliminary verification revealed no biases or asymmetries that would justify statistical corrections.

The analysis of biochemical parameters revealed that one‐way ANOVA showed no significant difference for urea levels (F(2.6) = 0.4184, *p* = 0.6759). On the other hand, significant differences were observed for creatinine (F(2.6) = 8.415, *p* = 0.0182), AST (F(2.6) = 8.173, *p* = 0.0194), and ALT (F(2.6) = 21.80, *p* = 0.0018). The Tukey test demonstrated a reduction in creatinine at the 2000 mg/kg dose (mean = 0.14) compared to the control (mean = 0.35). Conversely, the 2000 mg/kg dose induced a significant increase in AST levels (mean} = 129) relative to the control (mean = 29), as well as in ALT (mean = 68) compared to both the 300 mg/kg dose (mean = 37) and the control (mean = 36). The results are illustrated in Figure 4.

The results suggest that the ingestion of high doses of *C. sertanejus* EO is capable of altering biochemical parameters, thus indicating a need for caution. To the best of our knowledge, no studies have yet reported similar results involving EOs from species of the genus *Croton*. Despite these alterations, AST, ALT, and creatinine levels remain within the expected normal ranges for Swiss mice [40].

AST and ALT are two of the primary markers of liver integrity, and their elevations are commonly related to hepatocellular damage [41]. In contrast, creatinine serves as a key marker of renal function. Decreased serum creatinine levels have been associated with improved renal functional integrity, resulting from increases in glomerular filtration [42]. However, to support the biochemical parameter findings of the present study, histopathological examinations should be considered in future research.

## 3 Conclusion

Tests revealed that *C. sertanejus* EO exhibited significant spatial repellent activity against *Ae. aegypti* at concentrations starting from 1 mg/cm^2^. The effective concentrations for topically repelling 50% and 99% of the mosquitoes were 3 mg/cm^2^ and 109 mg/cm^2^, respectively. The 109 mg/cm^2^ concentration provided 64.8% protection against landings over 360 min and 100% protection against blood‐feeding over 150 min. The EO also demonstrated significant oviposition deterrence in water volumes of 20 mL (at concentrations starting from 2.5 mg/mL) and 500 mL (at concentrations starting from 0.005 mg/mL), but not in 1000 mL. Regarding the toxicity of *C. sertanejus* EO in *M. musculus*, no lethal dose was observed. However, at a dose of 2000 mg/kg, there was a significant reduction in brain weight, and at 300 mg/kg, an increase in lung and kidney weights was noted. At 2000 mg/kg, increased levels of aspartate aminotransferase and alanine aminotransferase, as well as a reduction in creatinine levels, also occurred. Regarding its chemical composition, the EO was characterized by a predominance of sesquiterpenes, although the monoterpene eucalyptol was identified as the major component.

Therefore, the *C. sertanejus* EO exhibits significant potential against *Ae. aegypti*. For continued studies, validation of the repellent action in vivo, the development of oviposition‐deterring formulations for use in larger water volumes, and a comprehensive toxicological assessment for practical application are recommended.

## 4 Experimental Section

### 4.1 Materials and Methods

#### 4.1.1 Plant Material

Leaves of *C. sertanejus* were collected in the Contendas do Sincorá National Forest (Bahia, Brazil) under SISBIO authorization no. (0670110420220208, 67011‐4). The collection was conducted in May 2023, selecting specimens under drought conditions and exposed to semi‐shade. ″Collection coordinates were recorded using a mobile GPS receiver (13°55′16.1″S, 41°07′51.1″W). The *C. sertanejus* specimen was identified by taxonomist Daniela Carneiro Torres and deposited in the Herbarium of the Federal University of Bahia (UFBA) under voucher number HVC6978. Specimen collection and research complied with national and international biodiversity guidelines, authorized by the Brazilian Genetic Heritage Management Council (CGEN/SisGen) under registration number AF28031.

#### 4.1.2 Essential Oil Extraction

The extraction of EO from *C. sertanejus* leaves followed the methodology described by Sousa et al. [12]. Briefly, the collected leaves were transported to the laboratory and stored at −18°C for preservation. Subsequently, the plant material was dried in a forced‐air oven at 45°C for 12 h until a brittle consistency was achieved. For the extraction process, 100 g of dried leaves was weighed and manually crushed to ensure a uniform particle size. The ground sample was transferred to a 2 L round‐bottom flask, to which 1.5 L of deionized water was added. The flask was then connected to a Clevenger‐type apparatus and placed in an appropriately sized heating mantle. The temperature was set to 100°C and maintained for 2 h, timed from the onset of reflux and vapor condensation.

At the end of the process, the EO was collected and the remaining aqueous phase (hydrolate) was removed using a polyethylene Pasteur pipette. To eliminate residual moisture, the EO was treated with anhydrous sodium sulfate (Na_2_SO_4_). Finally, the EO was transferred via micropipette to amber glass vials, which were hermetically sealed and stored at −4°C ± 1 until chemical analysis and bioassays were carried out.

#### 4.1.3 Chemical Composition of Essential Oils

The quantitative chemical composition was analyzed by gas chromatography coupled to a flame ionization detector (GC‐FID), using a CG 2010 Plus gas chromatograph (Shimadzu). Analyses were performed using a VF‐5 ms fused silica capillary column (30 m x 0.25 mm), with a 5% phenyl‐95% dimethylpolysiloxane stationary phase (0.25 *µ*m film thickness). Helium 6.0 was used as the carrier gas at a constant flow rate of 1.2 mL.min^−1^ (10 psi). The injector and detector temperatures were set at 250°C and 280°C, respectively. The injection volume was 0.1 *µ*L of CHCl3 solution at 50 ppm in a split ratio of 1:10. The column oven temperature was programmed at 50°C (held for 5 min), then ramped at 8°C/min^−1^ to 250°C and held for 5 min, totaling 36.25 min. Component quantification was performed by electronic integration of the FID peak areas using the normalization method.

Qualitative analysis was performed on a GCMS‐QP 2010 SE mass spectrometer (Shimadzu), equipped with a quadrupole mass analyzer. The chromatographic column and operating conditions were the same as those used in the GC‐FID analyses. The operating mode was electron ionization at 70 eV, with a scanning speed of 1 scan/s within a mass range of *m*/z 50 to 600 Da. The ion source and interface temperatures were set at 200°C and 250°C, respectively.

The EO components were identified based on their retention indices (RI). These indices were calculated for each compound by analyzing a homologous series of n‐alkanes (C8‐C26, Sigma‐Aldrich) under identical chromatographic conditions. The values were subsequently compared with those reported in the literature [18] and cross‐referenced with the mass spectral database (NIST 11).

### 4.2 Biological Assays

#### 4.2.1 Mosquito Collection and Formulations

The mosquitoes were obtained from a colony maintained at the Laboratory of Research and Natural Insecticides (LAPIN) of the State University of Southwestern Bahia (UESB), Itapetinga Campus. The specimens originated from eggs of the Rockefeller strain, kindly provided by the Laboratory of Physiology and Control of Arthropod Vectors (LAFICAVE) of the Oswaldo Cruz Foundation (FIOCRUZ‐RJ).

For each experiment, approximately 1,000 eggs were immersed in a polypropylene container (16.4 × 16.4 × 7.5 cm) containing 1 L of deionized water. Larvae were fed with 1 g of fish food (Alcon). The containers were maintained on laboratory benches in a climate‐controlled room at 27°C and 70% relative humidity, with daily monitoring. Upon pupation, specimens were transferred to polystyrene cups containing 70 mL of distilled water. To obtain adult mosquitoes, distinct methods were employed for each experiment, as detailed in their respective sections.

Regarding the preparation of the formulations for spatial repellency and oviposition deterrence, the EO of *C. sertanejus* was solubilized in Tween 80 using distilled water as a dispersant. Tween 80 was chosen as it effectively solubilized the EO in water. For topical repellency, the EO was formulated as a non‐ionic cream consisting of: Neolone (0.5%); polawax (10.5%); liquid petrolatum (4.74%); Tinogard TT (0.1%); Tinogard TL (0.05%); and distilled water q.s. ad (150 g). The non‐ionic cream was also used to formulate the analytical grade (p.a.) DEET positive control. The EO of *C. tetradenius* was also solubilized in Tween 80 and distilled water was used as a dispersant to evaluate oviposition deterrence.

#### 4.2.2 Spatial Repellency

The biological assay was performed according to the Guidelines for Efficacy Testing of Spatial Repellents of the World Health Organization (WHO) [19], using a horizontal two‐choice olfactometer adapted by Sousa et al. [12]. The apparatus consists of a central chamber and two lateral chambers covered with black oxford cloth. For the bioassay, five EO concentrations were tested: 5, 2.5, 1, 0.5, and 0.2 mg/cm^2^. Ten independent replicates of 20 mosquitoes each were performed per treatment, totaling 200 mosquitoes per concentration.

Following WHO guidelines, virgin female mosquitoes of the same age were used for the tests. In this study, specimens were obtained from isolated pupae in test tubes with approximately 1 mL of distilled water. Rearing took place in an room climate controlled at 27 ± 2°C and 80 ± 10% relative humidity. During the emergence period, the mosquitoes were transferred to separate cages (16.5 x 25 cm), grouped by emergence date, and fed daily with a 10% sucrose solution.

For experimental standardization, 7‐day‐old mosquitoes were used, which is an adequate period to ensure the required sample size. Twenty‐four hours prior to the experiment (at 6 days post‐emergence), 20 mosquitoes were transferred to six central chambers of the olfactometer, corresponding to each of the five EO concentrations (5, 2.5, 1, 0.5, and 0.2 mg/cm^2^) and the negative control (Tween 80). The transfer was performed using an adapted aspirator, and the mosquitoes remained in the olfactometer for acclimation until the following day. Feeding was suspended during this period to prevent heavy females, a factor that could interfere with the escape response to the treatments.

At the moment of the assay, the side chambers were connected to the central chamber system. With the olfactometer in a horizontal position, the bioassay was initiated as follows: with 20 mosquitoes in the central chamber, one side chamber was treated with 1 mL of the EO concentration applied uniformly to a strip of filter paper (26 x 76 mm) placed on a glass slide of the same size. The other side chamber was left untreated. Subsequently, the side chambers were wrapped with black oxford cloth. After one minute, the lids of the central chamber were opened simultaneously for ten minutes. Then, the lids were closed, and the mosquitoes inside the treated and untreated chambers were counted. These procedures were also applied to the negative control, which consisted of deionized water containing 5 mg/mL of Tween 80, equivalent to the highest concentration used for the EO.

To ensure the validity and statistical significance of the results, the number of mosquitoes per olfactometer and the number of replicates followed the WHO Guidelines for Efficacy Testing of Spatial Repellents [19]. The assays respected the diurnal habits of the species, occurring between 08:00 and 12:00, with two daily replicates executed. The trials were conducted in a climate controlled room at 27°C and 70% relative humidity. It is noteworthy that, for each replicate, different batches of mosquitoes aged up to seven days post‐emergence were used, as previously described.

### 4.3 Topical Repellent

#### 4.3.1 Estimation of the Effective Concentration

This assay aimed to estimate the effective concentrations (EC) capable of topically repelling 50% (EC_50_) and 99% (EC_99_) of the mosquitoes. To this end, this test followed the World Health Organization guidelines [20] and the methodology adapted by Sousa et al. [12], which consists of an in vitro evaluation utilizing *Capra aegagrus hircus* (Linnaeus, 1758) blood at 37°C as a chemical stimulus. The blood was supplied in a Glytube artificial feeder recommended by Costa da Silva et al. [21]. In the bioassay, five concentrations (125, 100, 75, 50, and 25 mg/cm^2^) were tested within a single cage, utilizing seven independent replicates of 50 mosquitoes each, for a total of 350 individuals.

The WHO suggests the use of mated females of uniform age. In the present study, mated females were obtained from cages containing both sexes. To achieve this, pupae reared in a climatically controlled room at 27°C and 70% relative humidity were transferred to cages in polyethylene cups containing 70 mL of distilled water. As the mosquitoes emerged, the cages were grouped by date, and a 10% sucrose solution was provided daily as food. To ensure experimental uniformity, mosquitoes with ten days of emergence were used a period considered adequate to reach the required sample size for the assays.

Twenty‐four hours prior to the experiment (on the ninth day of emergence), 50 female mosquitoes were transferred to the test cage (16.5 × 25 cm). This transfer was performed using an adapted aspirator, and the mosquitoes remained in the cage for acclimation until the following day. Feeding was suspended during this period to avoid interfering with blood‐seeking behavior during the assay

With the 50 mosquitoes inside the cage, five 4.9‐cm^2^ areas were marked on the top. A 0.1 g aliquot of the formulation, starting with the lowest concentration, was applied to one of the marked areas. After 1 min, an artificial feeder containing 1 mL of blood preheated to 37°C was placed over the treated area for 3 min. Evaluation criteria were based on the number of landings. The feeder was subsequently removed. This procedure was repeated for each subsequent step in increasing order of concentration. However, before testing the concentrations, the landing behavior of mosquitoes on the artificial feeder screen, both with and without the cream, was evaluated as a negative control.

The experiment was conducted over a single day (08:00 to 12:00) in a controlled environment (27°C and 70% relative humidity). To ensure the validity and statistical significance of the results, the number of mosquitoes per cage and the number of replicates followed the WHO guidelines [20]. The blood was collected from two male *Capra aegagrus hircus* at the Animal Science sector of the State University of Southwestern Bahia (UESB)—Itapetinga Campus, Bahia state, Brazil. The protocol was approved by the Institutional Animal Care and Use Committee of UESB (CEUA‐UESB; protocol no. 242/2024).

#### 4.3.2 Protection Time of the Effective Repellent Concentration

The methods for obtaining *Ae. aegypti* females for the EC protection time were the same as those described in the previous section. According to the Guidelines for Efficacy Testing of Mosquito Repellents for Human Skin [20], the protection time of the effective EC repellent concentration was evaluated at a concentration level of 99% (EC_99_). For this purpose, the methodology adapted by Sousa et al. [12] and the Glytube artificial feeder recommended by Costa da Silva et al. [21] were also utilized.

The bioassay was carried out using seven independent replicates of 50 mosquitoes per cage, totaling 350 individuals. In each replicate, an area of 4.9 cm^2^ was delineated on the netting at the top of the cage, where 0.1 g of the cream containing the EC_99_ was applied. After 30 min, the artificial feeder was exposed to the solution for three minutes. The feeder was then removed and placed in a water bath until the subsequent evaluation. This process was repeated at 30 min intervals over a total experimental period of 360 min.

The evaluation quantified landing repellency and the inhibition of blood feeding. Accordingly, protection time was defined as the elapsed time, in minutes, between repellent application and the initial landing and blood‐feeding. Previous records were used to verify the total number of landings and blood‐feedings at each evaluation interval. The identical procedures were applied to evaluate the control, positive, and negative groups. DEET served as the positive control, whereas the negative control consisted of the cage with and without cream.

The bioassays respected the diurnal habit of the species. However, for this assay, they were conducted between 08:00 and 13:00, with one daily replicate performed. The assays were also performed in an air‐conditioned room at 27°C and 70% relative humidity. It is worth noting that different batches of mosquitoes, up to seven days post‐emergence, were used for each replicate. The number of mosquitoes per cage and the number of replicates followed the WHO guidelines [20].

### 4.4 Oviposition Deterrence

#### 4.4.1 Assessment of the Concentration in a Smaller Volume of Water

The collection of females for the oviposition deterrence test followed the same procedure previously described in the “Estimation of the effective concentration” section. However, on the ninth day post‐emergence, 20 female mosquitoes were transferred to the test cages (16.5 x 25 cm). The transfer was performed using an adapted aspirator, and the mosquitoes were kept in the cage for acclimation until the following day. Feeding was suspended during this period to prevent interference with the next day's blood meal, which is essential for egg maturation.

To evaluate the EO of *C. sertanejus*, the methodology described by Santos et al. [22] and Soonwera and Phasomkusolsil was used, with minor modifications. Two concentrations (5 and 2.5 mg/mL) were tested in five independent replicates containing 20 mosquitoes each, totaling 100 mosquitoes per treatment.

For each concentration, 20 female mosquitoes were placed in a polypropylene cage (16.5 cm × 25 cm) and offered blood pre‐heated to 37°C via an artificial feeder for 1 h. Subsequently, a 10% sugar solution was provided inside the cage.Three days later, two 50 mL polystyrene containers, one with 20 mL of the solution at the target EO concentration, and the other with 20 mL of the negative control (deionized water and Tween 80 equivalent to the highest EO concentration) were placed in opposite corners inside the cage, containing cone‐shaped filter paper as an oviposition substrate. After 96 h (four days), the papers were removed, and the eggs were counted under a stereomicroscope.

The assay also considered the diurnal behavior of the species. Accordingly, the blood meal was offered between 08:00 and 12:00 for all replicates on the same day. The concentrations and respective replicates of the EOs were evaluated simultaneously on separate laboratory benches within the same climate‐controlled room where the mosquitoes were reared. To ensure greater rigor in the evaluation of oviposition deterrence, a larger number of mosquitoes per cage and a more prolonged evaluation period were employed compared to previously cited methodologies [22, 23]. The number of replicates was adopted in accordance with standard recommendations in the literature, thereby ensuring the validity and statistical significance of the data [22, 23].

#### 4.4.2 Evaluation in a Larger Volume of Water

Given that female *Ae. aegypti* select diverse oviposition sites, this study aimed to evaluate whether these mosquitoes can also detect the EO of *C. sertanejus* diluted in different water volumes. This approach allows for the establishment of minimum effective and economically viable concentrations, as the species can be induced to oviposit near the water surface in medium‐to‐large breeding sites [28].

To ensure greater rigor in the evaluation of oviposition deterrence, a larger number of mosquitoes per cage and a more prolonged evaluation period were employed compared to previously cited methodologies [22, 23]. The number of replicates was adopted in accordance with standard recommendations in the literature, thereby ensuring the validity and statistical significance of the data [22, 23].

Female mosquitoes were obtained following the same procedure described in the previous deterrence test. For the bioassay, 5 mg and 2.5 mg of the EOs were diluted to their respective volumes using Tween 80 as a surfactant at a 1:1 ratio. Diluting 5 mg of EOs in 0.5 and 1 L yielded concentrations of 0.01 mg/mL and 0.005 mg/mL, respectively. From the lower mass (2.5 mg), concentrations of 0.005 mg/mL and 0.0025 mg/mL were achieved. Three replicates were performed per concentration, totaling 60 mosquitoes per treatment. The evaluation protocol followed the previously described methodology, except for the oviposition cup removal time, which was adjusted to 90 h. The reduced number of replicates in this phase compared to the prior test was due to limited blood availability for the experiment. However, the experimental design was sufficient to ensure data validity and statistical significance.

#### 4.4.3 Preliminary Evaluation of the Acute Oral Toxicity of *Croton sertanejus* Essential Oil

Nine female Swiss mice (35 ± 2 g, aged 8–12 weeks) were used. The bioassay comprised three treatments: the EO at 300 and 2,000 mg/kg, and a 0.9% saline control group. Each treatment group consisted of three independent replicates, with *n* = 1 mouse per replicate, yielding a total of three mice per treatment.

The EO doses were administered orally, dissolved in a 0.9% saline solution (1 mL/kg). The control group received only 0.9% saline (1 mL/kg) following the Organization for Economic Co‐operation and Development (OECD) protocol 423 [24]. The number of animals used represents the minimum sample size required per treatment, in accordance with the OECD 423 guidelines.

Following a period of overnight fasting, the animals received the treatments and were individually housed in polypropylene cages.They were observed for the first four hours to detect any behavioral, cardiorespiratory, and gastrointestinal changes. Following this period, the animals were individually housed for daily monitoring of body mass, water intake, and food intake. On day 14, the animals were anesthetized (xylazine, 10 mg/kg, and ketamine, 100 mg/kg) for blood collection via venipuncture in the retro‐orbital plexus to measure serum levels of hepatic transaminases alanine aminotransferase (ALT) and aspartate aminotransferase (AST), urea, and creatinine.

Next, the animals were euthanized by deepening anesthesia (xylazine, 15 mg/kg and ketamine, 300 mg/kg, administered intraperitoneally) for the removal of organs and tissues, such as the brain, heart, lung, spleen, liver, kidney, uterus, abdominal adipose tissue, and retroperitoneal adipose tissue, which were subsequently weighed. Tibia length was used to normalize the organ and tissue weights, which is why the results were expressed in mg/mm. The experiment was conducted in a controlled environment (24°C and 60% relative humidity.

This stage of the study was conducted at UFPE in partnership with UESB. The protocol was approved by the Institutional Animal Care and Use Committee of UFPE (CEUA‐UFPE; protocol no. 0095/2023).

#### 4.4.4 Statistical Analysis

Bioassay data, with the exception of the EC_50_ and EC_99_ estimates for topical application, were initially tested for normality using the Shapiro‐Wilk test (*p *> 0.05) and for homogeneity of variances using Levene's test (*p *> 0.05). Data that violated either or both assumptions were subjected to normality adjustment via the bootstrapping procedure (1,000 resamples; bias‐corrected and accelerated [BCa] 95% confidence interval) and homogeneity correction via Welch's test. When data still did not meet assumptions after these adjustments, nonparametric tests were applied. Mosquito bioassays were analyzed using the IBM SPSS 21.0 software, while toxicity data for *M. musculus* were analyzed using GraphPad Prism 6. The specific statistical tests applied to each bioassay are detailed in the following.

#### 4.4.5 Spatial Repellent

The Spatial Activity Index (SAI) was calculated using the equation established by the World Health Organization [19]:

$$\mathrm{SAI}=\left[\left(\frac{\mathrm{Nc}-\mathrm{Nt}}{\mathrm{Nc}+\mathrm{Nt}}\right)\right]\times \left(\frac{\mathrm{Nm}}{N}\right)$$

Nc is the number of mosquitoes present in the tube without the formulation; Nt is the number of mosquitoes in the tube with the formulation; Nm is the total number of mosquitoes at the ends of the two tubes; and N is the total number of mosquitoes used in the test. The spatial activity index ranges from −1 to 1, where 0 indicates no response, −1 indicates non repellency, and 1 indicates a spatial repellent response.

The spatial activity index results were analyzed using a One‐Way ANOVA (*p* < 0.05). Normality was assessed using bootstrapping with 1,000 resamples and a bias‐corrected and accelerated (BCa) 95% confidence interval. Significant differences between means were identified using the Games‐Howell post‐hoc test (*p* < 0.05). The standard error of each index was calculated and considered in the analysis. The data were not transformed so as not to hinder comprehension.

### 4.5 Topical Repellent

#### 4.5.1 Estimation of the Effective Concentration for Topical Use

To estimate the effective concentration (EC) required to repel 50% (EC_50_) and 99% (EC_99_) of the mosquitoes, probit regression analysis was used at a 95% confidence level (*p* < 0.05). No adjustment procedure was required because the probit is a generalized linear model for binary data and does not rely on traditional linear assumptions [20].

#### 4.5.2 Protection Time of the Effective Concentration

The landing repellency means were subjected to a one‐way ANOVA (*p* < 0.05), with normality adjustment via bootstrapping (1,000 resamples, with a bias‐corrected and accelerated 95% confidence interval), and homogeneity correction via Welch's test. Mean comparisons were performed using the Games‐Howell post hoc test (*p* < 0.05). To evaluate protection against blood feeding, the non‐parametric Kruskal‐Wallis test (*p* < 0.05) was applied, followed by Dunn's post‐hoc test with Bonferroni correction (*p* < 0.05) to compare mean ranks. The means were also presented alongside the mean ranks to complement the interpretation of the results. The choice of a non‐parametric test in the blood meal evaluation was driven by limitations of the bootstrapping method on the data, likely caused by excess zeros. The data from both tests were not transformed, either, in order to preserve clarity.

#### 4.5.3 Oviposition Deterrence

The oviposition deterrent activity was expressed by the Oviposition Activity Index (OAI), using the formula established by Kramer and Mulla [25].

$$\mathrm{OAI}=\frac{\mathrm{NT}-\mathrm{Nc}}{\mathrm{NT}+\mathrm{Nc}}$$

where NT is the total number of eggs in the test solution and NC is the total number of eggs in the control solution. The oviposition activity index ranges from −1 to +1, in which 0 indicates no response. A positive index value indicates that more eggs were laid in the test solution, demonstrating no deterrence. Conversely, a negative index value indicates that fewer eggs were laid in the test solution, showing that it acts as a deterrent.

OAI means in a 20 mL water volume were compared using the independent‐samples *t*‐test (*p* < 0.05). A normality adjustment using the bootstrapping method (1,000 resamples, with a bias‐corrected and accelerated [BCa] 95% confidence interval) was also applied. The data were not transformed so as not to hinder comprehension.

Mean OAI values for the 500 and 1000 mL water volumes were compared using an unadjusted independent samples *t*‐test (*p* < 0.05). No parametric adjustment procedure was applied, as preliminary verification revealed no biases or asymmetries that would justify statistical corrections.

#### 4.5.4 Acute Toxicity

Results are expressed as the mean ± standard error of the mean (SEM). Water and food intake, as well as body weight over time, were evaluated using a two‐way repeated‐measures ANOVA, followed by Tukey's post hoc test (*p* < 0.05). Organ weights, adipose tissue, and biochemical parameters were analyzed using a one‐way ANOVA, also followed by Tukey's post hoc test (*p* < 0.05). No parametric adjustment procedure was applied, as preliminary verification revealed no biases or asymmetries that would justify statistical corrections.

## Author Contribution

**Daniel Lobo Sousa**: conceptualization, methodology, formal analysis, research, writing – original draft. **Morgana Maria do Carmo Barbosab**: investigation. **Rômulo Carlos Dantas da Cruz**: methodology. **Ivone Antonia de Souza**: methodology. **Rosilene Aparecida de Oliveira**: methodology. **Débora Cardoso da Silva**: methodology. **Simone Andrade Gualberto**: methodology. **Eduardo Carvalho Lira**: methodology and review. **Janaína Silva de Freitas**: methodology, resources, writing—review and editing, supervision.

## Funding

Fundação de Amparo a Pesquisa do Estado da Bahia (FAPESB‐ BOL0325/2022).

## Conflicts of Interest

The authors declare that they have filed a partial patent application for this manuscript.

## Data Availability

Data available on request from the authors.
